# Unbiased Identification of Proteins Covalently Modified by Complex Mixtures of Peroxidized Lipids Using a Combination of Electrophoretic Mobility Band Shift with Mass Spectrometry

**DOI:** 10.3390/antiox7090116

**Published:** 2018-08-30

**Authors:** Bernd Gesslbauer, David Kuerzl, Niko Valpatic, Valery N. Bochkov

**Affiliations:** Institute of Pharmaceutical Sciences, Department of Pharmaceutical Chemistry, University of Graz, Humboldtstrasse 46, 8010 Graz, Austria; david.kuerzl@edu.uni-graz.at (D.K.); niko.valpatic@edu.uni-graz.at (N.V.); valery.bochkov@uni-graz.at (V.N.B.)

**Keywords:** lipid oxidation, oxidized phospholipids, lipid-protein adducts, electrophoretic mobility shift assay, gel-shift, proteomics, mass spectrometry

## Abstract

Covalent modification of functionally important cell proteins by lipid oxidation products (LOPs) is a known mechanism initiating pathological consequences of oxidative stress. Identification of new proteins covalently modified by electrophilic lipids can be performed by a combination of chemical, immunological, and mass spectrometry-based methods, but requires prior knowledge either on the exact molecular structure of LOPs (e.g., 4-hydroxynonenal) or candidate protein targets. However, under the conditions of oxidative stress in vivo, a complex mixture of proteins (e.g., cytosolic proteome) reacts with a complex mixture of LOPs. Here we describe a method for detection of lipid-modified proteins that does not require an a priori knowledge on the chemical structure of LOPs or identity of target proteins. The method is based on the change of electrophoretic mobility of lipid-modified proteins, which is induced by conformational changes and cross-linking with other proteins. Abnormally migrating proteins are detected by mass spectrometry-based protein peptide sequencing. We applied this method to study effects of oxidized palmitoyl-arachidonoyl-phosphatidylcholine (OxPAPC) on endothelial cells. Several known, but also many new, OxPAPC-binding proteins were identified. We expect that this technically relatively simple method can be widely applied for label-free analysis of lipid-protein interactions in complex protein samples treated with different LOPs.

## 1. Introduction

Lipid oxidation is a characteristic feature of chronic inflammation associated with a variety of pathologies including, e.g., cardiovascular and neurodegenerative disease [1,2,3,4,5]. Oxidation of free or esterified polyunsaturated fatty acids (PUFAs) can be induced by reactive oxygen and nitrogen species or by multiple enzymes, generating a variety of bioactive compounds. In particular, oxidation of sn-2 PUFA residues in phospholipids (PLs) generates a variety of full-length and truncated reactive oxidized PLs (OxPLs), which are increasingly recognized for their pleiotropic biological action including proinflammatory and prothrombotic effects, as well as stimulation of angiogenesis and protection of lung endothelial barrier [6]. OxPLs are known to be present in blood and tissues; their total concentrations in pathological conditions can reach micromolar range, while in atherosclerotic vessels total concentrations of several molecular species may be in a high micromolar range [1]. Lipid oxidation products (LOPs) containing electrophilic terminal aldehyde or α,β-unsaturated carbonyl groups can react with nucleophilic groups of biomacromolecules, such as thiol and amino groups of proteins, forming covalent Michael adducts or Schiff bases. Adduction of LOPs to proteins can alter protein structure and function, and consequently influence cellular signal transduction pathways [1,7,8]. This covalent modification of proteins may be quite selective, for example, modification of the β-subunit of IκB kinase prevents IκBα degradation and NF–κB activation [9]. Therefore, the knowledge of protein targets of LOPs is a prerequisite for better understanding of mechanisms of oxidative stress.

Several approaches to identify protein targets of LOPs that have been developed in the last years can be divided in two groups. The majority of studies identified proteins in crude preparations, e.g., cytosolic proteins, that were modified by a specific chemically-pure LOP, e.g., HNE (4-hydroxynonenal). Another group of publications analysed specific amino acid residues within a defined protein, which may be modified by one or different reactive LOPs. The most extensively investigated lipid-derived electrophile is HNE. Identification of HNE-protein adducts has been performed mainly by following three approaches: (i) tandem mass spectrometry (MS/MS and MS^3^)-based detection of HNE-modified peptides [10,11]; (ii) immune detection of HNE-protein adducts by Western blotting followed by liquid chromatography tandem mass spectrometry (LC-MS/MS)-based identification of immunoreactive protein bands [12,13,14]; and (iii) attachment of biotin through click chemistry and enrichment of modified proteins or peptides using immobilized streptavidin. A variety of carbonyl-reactive probes have been developed to capture HNE-modified proteins and other carbonyl-containing compounds. These probes contain typically hydrazide or aminooxy functional groups, which are conjugated with biotin for streptavidin-based enrichment [15,16,17,18,19]. In addition to these post-labeling strategies, synthetic alkynyl- and azido-derivatives of HNE have been used to treat cells or isolated proteomes followed by biotin/streptavidin-based purification of modified proteins [20,21]. Site-specific characterization of cysteine modifications by different LOPs using thiol-specific probes was reported by Wang et al. [22]. With this chemoproteomic method, which is based on the quantification of unmodified cysteines (because LOP-modified cysteines are protected from thiol-specific labelling), >1000 reactive cysteines were quantified in isolated proteomes.

In contrast to the relatively well investigated protein targets of HNE, proteomic approaches to identify OxPL-protein adducts, are rather limited, because the enrichment methods that are reported for HNE-protein adducts cannot be directly applied to OxPLs.

Gugiu et al. used biotinylated 1-palmitoyl-2-arachidonoyl-*sn*-glycero-3-phosphoethanolamine (PAPE), which was oxidized on air to generate biotin-labeled OxPAPE (OxPAPE-N-biotin), to identify OxPAPE protein targets from human aortic endothelial cells [23]. Biological activity of OxPAPE-N-biotin was proven by upregulation of mRNA of inflammatory genes. Biotinylated OxPAPE-protein adducts were enriched with avidin beads, separated by sodium dodecyl sulfate polyacrylamide gel electrophoresis (SDS-PAGE) and detected by Western blotting using HRP-streptavidin (horseradish peroxidase-streptavidin). Protein bands containing biotinylated adducts detected by Western blot analysis were cut from an SYPRO Ruby stained gel and subjected to mass spectrometric identification. Overall, 29 mainly cytosolic and mitochondrial proteins could be identified from positively-stained protein bands, which were proposed as potential targets of OxPAPE in endothelial cells.

A similar approach was reported by Tallman et al. using a biotin-modified 1-palmitoyl-2-linoleoyl-*sn*-glycero-3-phosphatidylcholine (PLPC) probe to identify protein targets of OxPLs in human plasma [24,25]. In contrast to Gugiu et al., the eluate from streptavidin column was directly analysed by mass spectrometry (MS) resulting in the identification of 21 plasma proteins representing potential targets of this synthetic PC.

Fluorescently-labelled OxPL species represent a further method to detect OxPL-protein adducts. Stemmer et al. used synthetic BODIPY-labeled 1-palmitoyl-2-(5-oxovaleroyl)-*sn*-glycero-phosphoethanolamine to identify OxPL target proteins from mouse macrophages [26]. Proteins were separated by 1D and 2D gel electrophoresis followed by in-gel digestion and MS/MS based identification of proteins present in fluorescent protein spots/bands.

Multistep reversed phase C18-solid phase extraction enrichment in combination with aminolysis was applied by Gao et al. to enrich OxPL-modified peptides from unmodified peptides based on differences in hydrophobicity [27]. In this study, human platelets were incubated with liposomes containing 9-keto-12-oxo-10-dodecenoate-PC (KODA-PC) followed by cell lysis and tryptic in-solution digestion. Resulting peptides were first fractionated by C18-SPE into hydrophobic OxPL-modified peptides and hydrophilic peptides. Afterwards, hydrophobic OxPL-modified peptides were subjected to aminolysis, resulting in cleavage of the oxidized fatty acid at sn-2 position from the backbone. The resulting peptide-lipid electrophile adducts, which are much more hydrophilic than their precursors, were finally separated by C18-SPE from other hydrophobic compounds. MS/MS-based identification of peptide-lipid electrophile adducts resulted in the identification of 6 KODA-PC-modified proteins.

The abovementioned methods allowed the identification of a variety of potential targets of OxPLs. However, all these approaches have some drawbacks because they are either quite biased, since only a single LOP or a very small set of well-defined LOPs can be used, or require labelling or chemical modification of LOPs. Label-free identification of LOP-protein/peptide adducts using MS/MS requires knowledge of the exact structure of the LOP used. Furthermore, protein identification is often based on only one or two sequenced LOP-modified peptides. Antibody-based detection of unlabelled LOP-protein complexes from gels may result in false-positive hits, since not all proteins identified from a single spot/band are modified. Labelling or chemical modification for click chemistry may alter intrinsic properties of LOPs and, thus, may influence reactivity, selectivity and specificity of binding.

Here we report a novel straightforward approach to identify protein targets of OxPLs without labelling of the lipids or chemical reactions after the binding has occurred. The method is based on MS detection of changes in electrophoretic mobility of proteins covalently modified with electrophilic OxPLs as compared to intact proteins. The method is in principle independent of the general chemical nature of electrophilic LOPs (e.g., free or esterified), chemistry of covalent protein modification (e.g., Michael adduction, Schiff base formation), complexity of the LOP-sample (e.g., individual molecular species or complex mixtures such as OxPAPC, OxPAPE, oxidized palmitoyl-arachidonoyl-phosphatidylserine, etc.) or proteome (e.g., a pure protein, cell lysate, membrane-associated proteins, etc.). In other words, this is a technically straightforward, universal and unbiased method. Here we describe the application of this method for identification of OxPL-binding proteins in cytosolic and membrane fractions of endothelial cells.

## 2. Materials and Methods

Unless otherwise specified, all reagents were from Sigma-Aldrich (St. Louis, MO, USA).

### 2.1. Lipids and Cell Culture

1-Palmitoyl-2-arachidonoyl-*sn*-glycero-3-phosphocholine (PAPC) was obtained from Avanti Polar Lipids (Alabaster, AL, USA) and oxidized by prolonged exposure of pure lipid to air [28]. The extent of oxidation was monitored by positive ion electrospray MS.

Human umbilical vein endothelial cells (HUVEC) immortalized by telomerase overexpression were kindly provided by Dr. Hannes Stockinger (Medical University of Vienna, Austria). The cells were cultured in Nunclon Delta T75 flasks or six-well plates (Thermo Fisher Scientific, Waltham, MA, USA) in medium 199 (Gibco, Carlsbad, CA, USA) supplemented with 20% foetal calf serum, penicillin-streptomycin-amphotericin B (Lonza, Basel, Switzerland), and ECGS-heparin (PromoCell, Heidelberg, Germany) in the 95% humidified atmosphere with 5% CO_2_ at 37 °C. For protein isolation, 90% confluent HUVECs in flasks were washed three times with 10 ml of phosphate buffered saline (PBS) followed by harvesting in PBS containing cOmplete™ Protease Inhibitor Cocktail (PBS-PI) using a scraper. Cells were pelleted by centrifugation at 450× *g* for 10 min.

For Western blotting, HUVECs were cultured until 80% confluence in six-well plates. Prior to OxPAPC treatment, cells were washed with serum-free medium. OxPAPC in PBS (or the same volume of PBS without OxPAPC) was added to a final concentration of 80 µmol/L. Cell medium was removed immediately or after 6 h followed by cell scraping in PBS-PI. Cells were pelleted by centrifugation at 450× *g* for 10 min and resulting cell pellets were resuspended in RIPA buffer (radioimmunoprecipitation assay buffer) containing protease inhibitors (RIPA-PI). Cell lysis was performed by sonication on ice.

### 2.2. Isolation of HUVEC Proteomes 

Cells harvested from 10 T75 flasks were pooled, resuspended in 5 mL ice-cold PBS-PI and lysed by sonication on ice. After incubation of the suspension at 4 °C for 30 min with gentle shaking, PBS-insoluble material was pelleted by centrifugation at 17,000× *g* for 40 min. The supernatant containing “PBS-soluble proteins” was transferred to a new vial. The remaining pellet was washed five times with 5 mL of PBS-PI followed by centrifugation at 17,000× *g* for 40 min. The supernatant was discarded, the pellet redissolved in RIPA buffer and incubated at 4 °C for 30 min with gentle shaking. RIPA buffer enables efficient cell lysis and solubilization of membrane proteins while avoiding the loss of protein immunoreactivity. The final solution is composed of 150 mmol/L NaCl, 1.0% (*v/v*) IGEPAL^®^ CA-630, 0.5% (*w/v*) sodium deoxycholate, and 0.1% (*w/v*) SDS (sodium dodecyl sulfate), 50 mmol/L Tris, pH 8.0. After centrifugation at 17,000× *g* for 40 min, the supernatant (“PBS-insoluble/RIPA-soluble proteins”) was transferred to a new vial. Protein concentration was determined using the Pierce™ BCA Protein Assay Kit (Thermo Fisher Scientific).

### 2.3. Electrophoretic Mobility Band Shift Analysis (EMSA)

OxPAPC treatment of isolated proteomes was performed with 12 µg of protein in PBS-PI or RIPA-PI buffer with varying OxPAPC concentrations ranging from 40–100 µmol/L in a final volume of 30 µL. Samples were incubated at 37 °C with gentle shaking. Formation of OxPL-protein adducts was stopped by addition of 10 µL 4× Laemmli buffer (250 mmol/L Tris-HCl, pH 6.8, 40% (*v/v*) glycerol, 8% (*w/v*) SDS, 0.02% (*w/v*) bromophenol blue, and 10% (*v/v*) 2-mercaptoethanol) after 0 or 90 min followed by heating for 5 min at 95 °C. Band shift analysis was performed by standard SDS-PAGE using commercial 4–20% TGX gels (Bio-Rad Laboratories, Hercules, CA, USA) or homemade 10% acrylamide Tris/glycine gels (composition of the separating gel: 10% (*v/v*) acrylamide/bis-acrylamide (37.5:1), 375 mmol/L Tris-HCl, pH 8.8, 0.1% (*w/v*) SDS, 0.033% (*w/v*) ammonium persulfate, and 0.066% (*v/v*) tetramethylethylenediamine; composition of the stacking gel: 3.9% (*v/v*) acrylamide/bis-acrylamide (37.5:1), 125 mmol/L Tris-HCl, pH 6.8, 0.1% (*w/v*) SDS, 0.05% (*w/v*) ammonium persulfate, and 0.1% (*v/v*) tetramethylethylenediamine). Electrophoresis was performed with the Mini-Protean Tetra System (Bio-Rad) using a Tris/glycine/SDS running buffer (25 mmol/L Tris, 192 mmol/L glycine, 0.1% (*w/v*) SDS, pH 8.3) and keeping the current constant at 15 mA per gel. A total amount of 5 µg of protein in Laemmli buffer was applied to each slot. Visualization of protein bands was done by the EMBL silver staining protocol [29]. Proteins from samples incubated for 90 min with and without OxPAPC were selected for proteomics-based identification. To this end, the whole gel (above a molecular weight of ~15 kDa) was cut into multiple pieces with special attention to cut gel pieces of treated and untreated samples at exactly the same position.

### 2.4. In-Gel Protein Digestion and Nano-LC-MS/MS Analysis

For in-gel digestion protein bands were excised, washed for 10 min each with 200 µL 50 mmol/L NH_4_HCO_3_ and NH_4_HCO_3_: acetonitrile 1:1, followed by dehydration with acetonitrile (HPLC grade, VWR, Radnor, PA, USA). Each sample was then subjected to reduction with 100 µL of a 50 mmol/L NH_4_HCO_3_ solution, containing 10 mmol/L dithiothreitol at 54 °C for 30 min, and to alkylation with 150 µL of a 50 mmol/L NH_4_HCO_3_ solution, containing 50 mmol/L iodoacetamide for 20 min at room temperature in the dark. The gel slices were then washed again with 50 mmol/L NH_4_HCO_3_ followed by washing with acetonitrile and drying in a vacuum centrifuge. For in-gel digestion, the gel slices were rehydrated in 15 µL of a trypsin solution (5 ng/µL; sequencing grade Trypsin, Roche, Basel, Switzerland) in 50 mmol/L NH_4_HCO_3_ and incubated overnight at 37 °C. Peptides were extracted from the gel matrix with 25 µL 50 mmol/L NH_4_HCO_3_ and subsequently two times with 25 µL of 5% (*v/v*) formic acid in an ultrasonic bath. All supernatants were combined and analysed by nano-LC-MS/MS.

Nano-LC separations were performed on an UltiMate 3000 RSLCnano system (Thermo Fisher Scientific, Waltham, MA, USA). The flow rate of the nano-HPLC system was set at 250 nL/min and the UV absorbance was detected at λ = 214 nm. The flow rate of the loading pump was set at 20 µL/min. The trap column dimensions were 300 µm id × 5 mm length, packed with PepMap C18 (Thermo Fisher Scientific, Waltham, MA, USA). After a sample loading time of 10 min, the trap column was switched in line with the nanocolumn. The sample was eluted in back flush mode. The mobile phases were: (A) 99.9% (*v/v*) water (HPLC grade, VWR) and 0.1% (*v/v*) formic acid; and (B) 80% (*v/v*) acetonitrile (HPLC grade, VWR) and 0.08% (*v/v*) formic acid. The mobile phase for the loading pump was water with 0.05% (*v/v*) trifluoroacetic acid. The HPLC gradient for separation was 4% B for 10 min, 4–40% B in 90 min, and 40–90% B in 5 min, 90% B for 5 min, 4% B for 20 min. For separation an Acclaim PepMap RSLC column (C18, 75 µm × 150 mm, 2 µm, 100 Å; Thermo Fisher Scientific, Waltham, MA, USA) was used. Eluted peptides were ionized via stainless steel emitters using a Nanospray Flex™ ion source (Thermo Fisher Scientific) and directly introduced into a LTQ XL mass spectrometer (Thermo Fisher Scientific).

The following electrospray ionization parameters were used: spray voltage, 1.7 kV; capillary temperature, 200 °C; capillary voltage, 30 V. The collision energy was set automatically depending on the mass of the parent ion. The data were collected in the centroid mode using one MS experiment followed by five MS/MS experiments of the most intensive ions (intensity at least 5 × 10^3^). Dynamic exclusion was used for data acquisition with exclusion duration of 2 min and an exclusion mass width of ±1.5 Da.

For peptide identification, RAW-files were converted into MGF-files using ProteoWizard [30] and analysed with the MASCOT search engine (Matrix Science, London, UK). All MS/MS spectra were searched against the SwissProt protein sequence database. Following search parameters were used: carbamidomethylation on cysteine was set as a fixed modification; oxidation on methionine was set as a variable modification; trypsin was set as enzyme; the precursor mass tolerance was set to 3 Da; the fragment mass tolerance was set to 0.8 Da; the maximal number of missed cleavages was set to 2. The results were filtered to peptide scores ≥30 and to a 1% false discovery rate using Mascot. Representative MS/MS spectra of certain OxPAPC modified proteins can be found in Supplementary Material (Figures S2–S6). To calculate protein abundance of all proteins in a particular proteome fraction based on Exponentially Modified Protein Abundance Index (emPAI) score [31], MGF-files of all samples of a particular fraction were merged and analysed with the MASCOT search engine.

### 2.5. Data Analysis

Mascot search results from all single bands from treated and untreated samples were compared. A protein was regarded as modified by OxPAPC, if the corresponding protein was found in the primary band of both, OxPAPC treated and untreated samples, as well as in higher molecular weight regions of OxPAPC-treated samples compared to control samples. At least five unique peptides of each modified protein had to be identified in higher molecular weight regions.

### 2.6. Protein-Protein Interaction Network Construction and Functional Annotation 

To analyse protein-protein interaction networks and to perform functional annotation of identified proteins, the Search Tool for the Retrieval of Interacting Genes (STRING) database was used.

### 2.7. Western Blotting

For Western blotting, samples from OxPAPC-treated live cells (see Section 2.1) and samples from gel band shift analysis (see Section 2.3) were used. SDS-PAGE was performed as described in Section 2.3. Proteins were transferred by semi-dry blotting to a BioTrace NT nitrocellulose membrane (Pall Corporation, Port Washington, NY, USA) using the Trans-Blot Turbo System (Bio-Rad) for 30 min keeping the voltage constant at 25 V. Afterwards the membrane was incubated in Tris-buffered saline, 0.05 % (*v/v*) Tween 20 (TBST) containing 3% (*w/v*) BSA for 60 min. Primary antibodies diluted in TBST containing 1% (*w/v*) BSA, were added to the membrane and incubated over night at 4 °C with gentle shaking. After three washing steps for 5 min with TBST, the membrane was incubated with horse radish peroxidase (HRP)-linked secondary antibodies for 60 min. After five washing steps for 5 min, the membrane was incubated with the Clarity ECL substrate (Bio-Rad) for 4 min. Chemiluminescence imaging was performed using the G:BOX Chemi XX6 (Syngene, Frederick, MD, USA). The following primary antibodies and corresponding dilutions were used: anti-HSP90β (anti-heat shock protein HSP 90-beta) (ADI-SPA-844-050; Enzo Life Sciences, Farmingdale, NY, USA), 1:1000 dilution; anti-β-Actin (A2103; Sigma-Aldrich, St. Louis, MO, USA) 1:1000 dilution; anti-BAP31 (B-cell receptor-associated protein 31), anti-Integrin α2, anti-CD13, anti-VDAC1 (sc-393810, sc-74466, sc-166105, sc-390996; Santa Cruz Biotechnology, Dallas, TX, USA) all diluted 1:500. The following secondary antibodies and corresponding dilutions were used: HRP-linked Anti-rabbit IgG (7074; Cell Signalling Technology, Danvers, MA, USA), 1:3000 dilution; or HRP-linked mouse-IgGκ binding protein (sc-390996; Santa Cruz Biotechnology, Dallas, TX, USA), 1:8000 dilution.

## 3. Results

### 3.1. Incubation with OxPLs Changes the Electrophoretic Mobility of Certain Proteins

**Figure 1:**
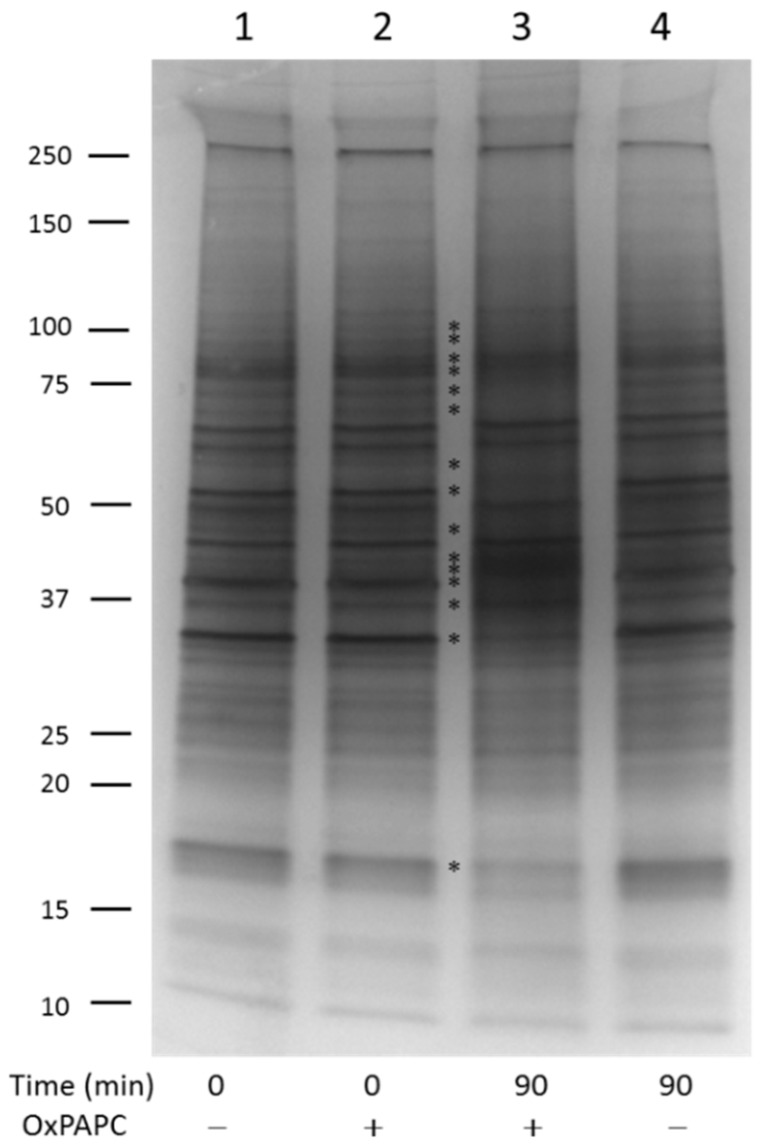
Gel band shift analysis of PBS-soluble human umbilical vein endothelial cell (HUVEC) proteins. Oxidized palmitoyl-arachidonoyl-phosphatidylcholine (OxPAPC) incubated with proteins for 90 min significantly changed the electrophoretic protein profile (lane 3). OxPAPC which is not covalently bound to proteins does not influence the electrophoretic mobility of proteins (lane 2). Lane 1: control sample time point 0; lane 4: control sample time point 90. The final OxPAPC concentration in treated samples was 100 µmol/L. Main visible changes are marked with asterisks.

In order to reduce the complexity of the proteome and to separate water-soluble, mainly cytosolic proteins, from membrane-associated proteins, we split the HUVEC proteome into a PBS-soluble part and a PBS-insoluble/RIPA-soluble part. Both samples were subjected to EMSA after treatment with varying concentrations of OxPAPC. If OxPAPC was not covalently bound to proteins (i.e., at time 0), the electrophoretic mobility of PBS-soluble proteins was not influenced (Figure 1, lane 2 compared with control lane 1). Incubation of the proteome at 37 °C for 90 min without OxPAPC, likewise did not influence the electrophoretic mobility of proteins (Figure 1, lane 4). However, if the sample was incubated with OxPAPC for 90 min, clear changes, including band shifts, band-smearing, and intensity changes can be observed (Figure 1, lane 3).

**Figure 2:**
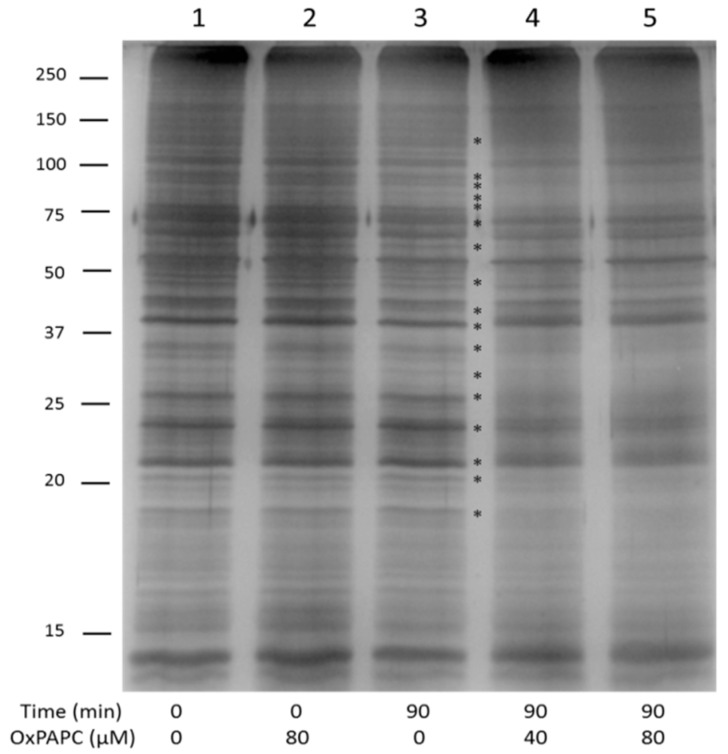
Gel band shift analysis of PBS-insoluble/RIPA-soluble HUVEC proteins. OxPAPC incubated with proteins for 90 min significantly changed the electrophoretic protein profile (lanes 4 and 5). OxPAPC which is not covalently bound to proteins does not influence the electrophoretic mobility of proteins (lane 2). Lane 1: control sample time point 0; lane 3: control sample time point 90. The final OxPAPC concentration in treated samples was 40 and 80 µmol/L. Main visible changes are marked with asterisks.

These OxPAPC-induced changes of the electrophoretic protein profile were also observed within the PBS-insoluble/RIPA-soluble proteome (Figure 2). Similarly to the experiment with hydrophilic proteins, unbound OxPAPC did not influence the electrophoretic mobility of hydrophobic proteins (Figure 2, lane 2 compared with control lane 1). Treatment of proteins with two different concentrations of OxPAPC for 90 min induced significant changes such as band shifts, band smearing, and intensity changes (Figure 2, lanes 4 and 5).

The band shift assay was performed using several independent protein preparations from HUVECs at different passages. In all cases we observed clearly visible and reproducible changes of the electrophoretic protein profile in silver-stained gels (data not shown). Since each visible band contained multiple proteins, it was impossible to reliably identify OxPAPC-modified proteins by targeted excision and sequencing of bands that became weaker or stronger upon OxPAPC treatment. Therefore, we applied an alternative approach, namely cut the whole gel into pieces and identified position of each protein in the gel as described in the next paragraph. The method successfully identified OxPAPC-modified proteins due to their abnormal migration in the gel.

### 3.2. Protein Targets of OxPAPC Identified by LC-MS/MS

**Figure 3:**
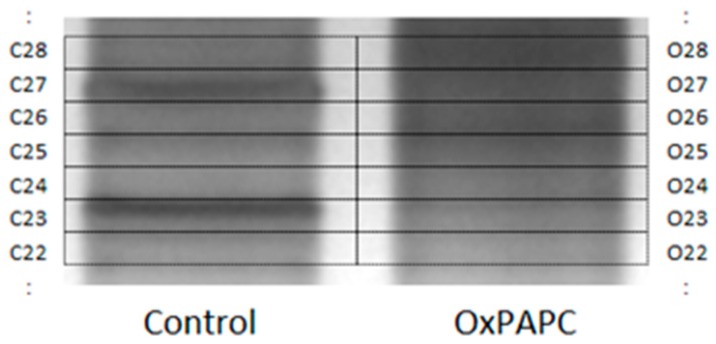
Representative region of a gel showing changes in pattern of protein silver staining. The grid shows how the bands were excised for in-gel trypsinisation and LC-MS/MS (liquid chromatography tandem mass spectrometry) analysis.

In order to identify proteins that changed their electrophoretic mobility and thus represent targets of OxPAPC, protein bands from samples incubated with and without OxPAPC were analyzed by nano-LC-MS/MS. The whole gel (above a molecular weight of ~15 kDa) was cut into multiple pieces with special attention to cut gel pieces of treated and untreated samples at exactly the same position (Figure 3).

The bands were subjected to in-gel digestion and resulting peptides were identified by LC-MS/MS. Comparison of proteins identified from individual bands from treated and untreated samples clearly showed that in OxPAPC-treated samples several proteins shifted from their major band location to positions in higher molecular weight regions. A protein was regarded as modified by OxPAPC, if the corresponding protein was found in (i) the primary band of both samples and (ii) in higher molecular weight regions of OxPAPC-treated samples compared to control samples.

From the PBS-soluble fraction, 21 proteins were shifted to higher molecular weight regions in OxPAPC treated samples and thus regarded as modified by OxPAPC (Table 1). Since in total over 500 proteins were identified in both control and OxPAPC-treated samples, ~4% of the detected proteome was identified as modified.

In the PBS-insoluble/RIPA-soluble fraction 37 proteins were identified as potential targets of OxPAPC (Table 2). In this proteome, over 700 proteins could be detected both in control and OxPAPC-treated samples, thus ~5% were detected as modified.

**Figure 4:**
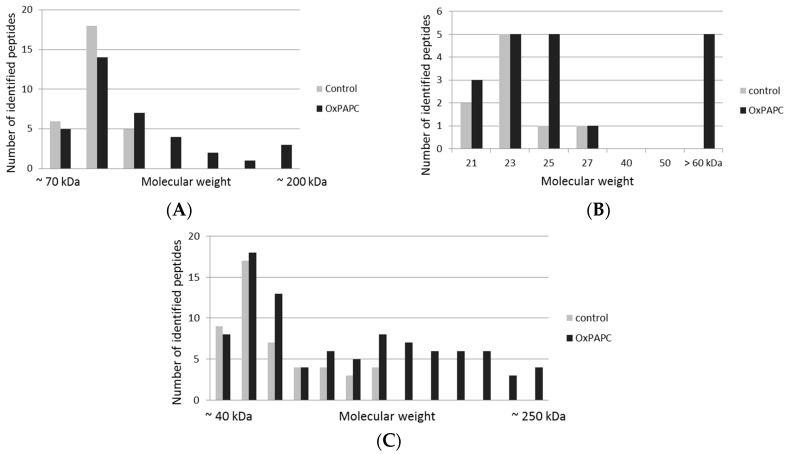
Peptide distribution of selected representative OxPAPC-modified proteins. A clear increase of peptides identified in higher molecular weight regions is observed after OxPAPC treatment. (**A**) Peptide distribution of HSP 90-beta; (**B**) Peptide distribution of VDAC-1; (**C**) Peptide distribution of Actin, cytoplasmic 1.

The number of identified peptides of a modified protein was plotted against the estimated molecular weight region on the gel, where the protein was detected. The distribution of peptides corresponding to individual proteins in gel bands cut out in different gel regions is shown in Figure 4. The examples include voltage-dependent anion-selective channel protein 1 (VDAC-1), heat shock protein HSP 90-beta (HSP90β), and actin cytoplasmic 1 (β-actin). A clear increase of the number of peptides identified in higher molecular weight regions was observed in OxPAPC-treated samples.

**Figure 5:**
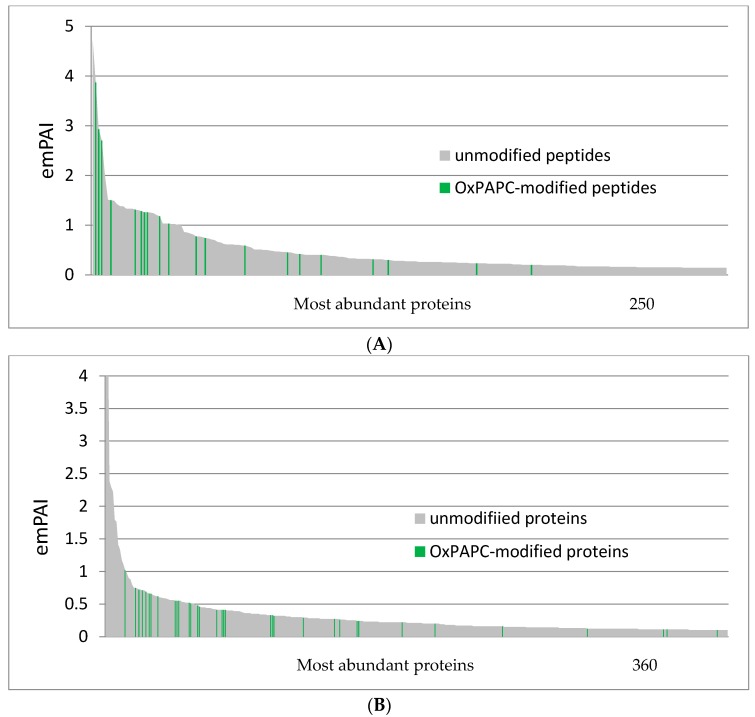
Protein abundance, based on exponentially modified protein abundance index (emPAI) score. Most abundant proteins, representing ~85% of total proteins, were plotted. The property of proteins to be modified by OxPAPC is not a simple function of their abundance. (**A**) PBS-soluble proteins; and (**B**) PBS-insoluble/RIPA-soluble proteins.

To investigate if proteins modified by OxPAPC represent only highly abundant proteins in the particular proteome, the Exponentially Modified Protein Abundance Index (emPAI), which is automatically calculated by Mascot, was used. The MS/MS RAW-data of all protein bands of a particular proteome fraction were merged and searched against the SwissProt protein sequence database using Mascot. emPAI scores of the most abundant proteins, representing together ~85% of total proteins, are shown in Figure 5. The analysis was performed separately for soluble and membrane RIPA-soluble proteins. In the PBS-soluble fraction, several highly abundant proteins were found to be modified by OxPAPC (Figure 5A), while the most highly abundant proteins from the RIPA-soluble fraction formed no adducts with OxPAPC (Figure 5B). Nevertheless, the majority of highly abundant proteins was not modified in both fractions and vice versa. Additionally, low abundance proteins were modified in both proteomes. The data suggest that the property of proteins to be modified by OxPAPC is not a simple function of its abundance.

**Figure 6:**
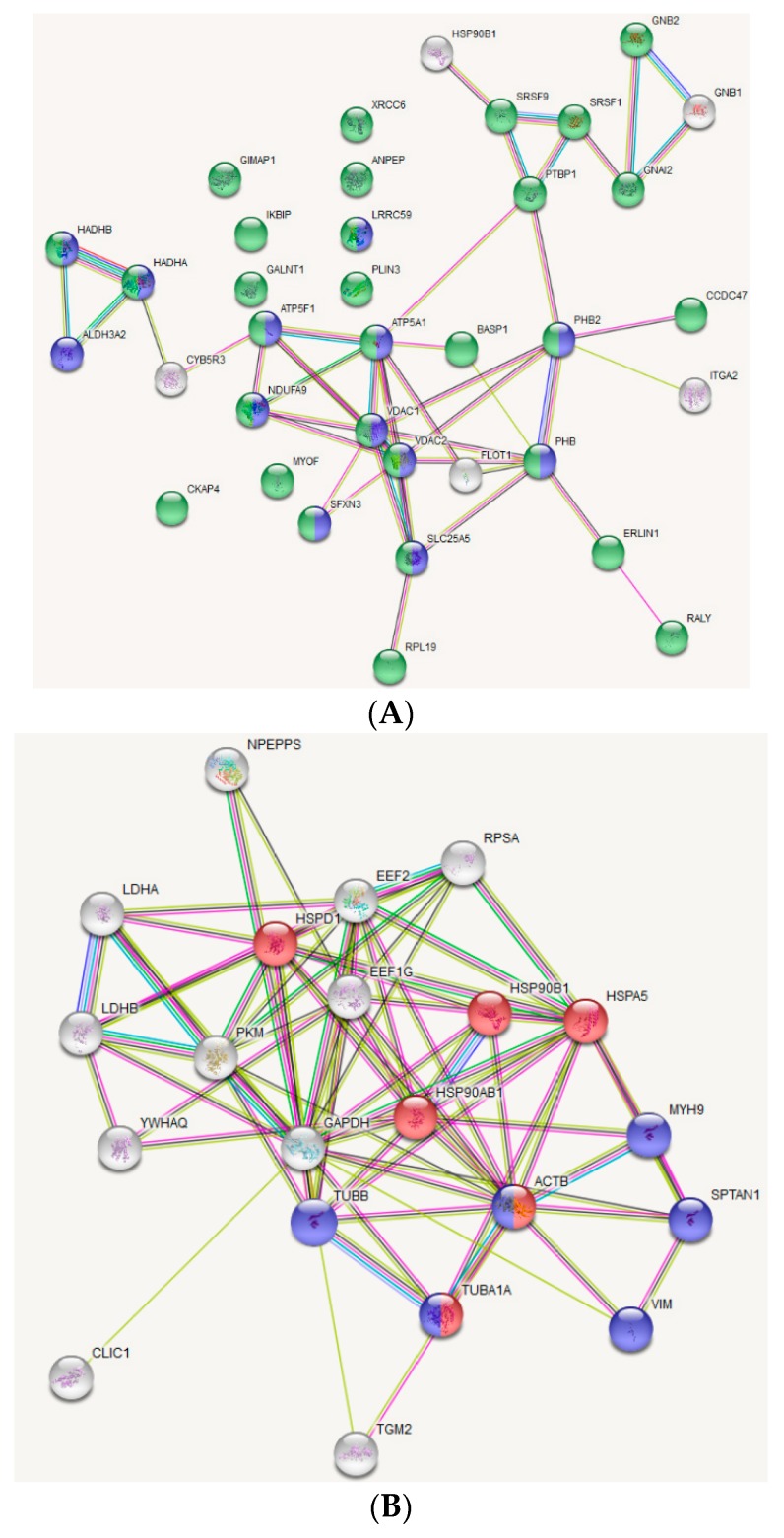
STRING analysis of proteins identified as targets of OxPAPC from the PBS-insoluble/RIPA-soluble fraction (**A**) and from the PBS-soluble fraction (**B**). The RIPA-soluble fraction of modified proteins contains mainly proteins located at membrane organelles (green nodes: membrane-bounded organelle; GO: 0043227). Furthermore, several modified proteins are located at the mitochondrial membrane (blue nodes: mitochondrial part; GO: 0044429). In the PBS-soluble fraction, proteins related to protein folding (red nodes: protein folding; GO: 0006457) and proteins related to the cytoskeleton (blue nodes: structural constituent of cytoskeleton; GO: 0005200) are enriched.

STRING analysis of OxPAPC-modified proteins from the RIPA-soluble fraction showed a clear enrichment of proteins located at membrane organelles in general (Figure 6A; green nodes) and proteins located at the mitochondrial membrane (Figure 6A; blue nodes) in particular. Furthermore, these mitochondrial proteins form a tight protein-protein interaction network. STRING analysis of the PBS-soluble fraction showed an enrichment of proteins related to protein folding (Figure 6B; red nodes) and proteins representing structural constituents of the cytoskeleton. Based on these results, mitochondrial (membrane-) proteins, proteins involved in protein folding and cytoskeletal proteins, seem to be major targets of OxPAPC.

### 3.3. Confirmation of OxPAPC-Induced Protein Modifications by Western Blotting

**Figure 7:**
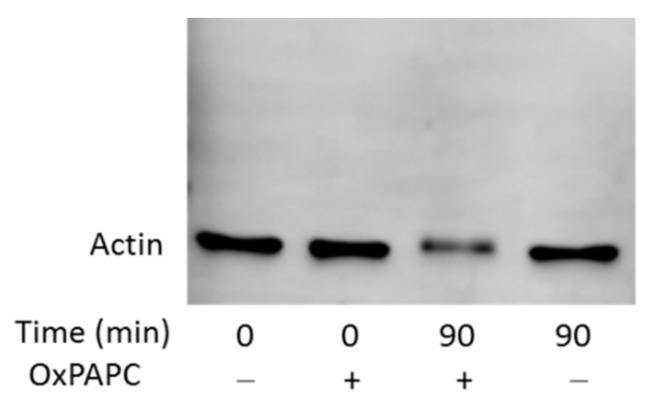
Western blot analysis of β-Actin from the OxPAPC-treated PBS-soluble fraction. The OxPAPC-treated sample incubated for 90 min, shows a decrease of actin immunostaining and a slightly slower band migration. The final OxPAPC concentration was 100 µmol/L.

To confirm that the apparent abnormal migration of OxPAPC-treated proteins in the gel was not an artifact of MS detection, we performed western blot analysis of selected proteins to detect these proteins by antibodies. Since β-actin is a rather highly abundant protein, which was identified to be modified by OxPAPC in the PBS-soluble fraction, we used a polyclonal anti-β-Actin antibody to analyse OxPAPC-actin adducts. We could observe a significant decrease of the amount of β-actin at its major band, as well as a slight band shift of actin in OxPAPC treated samples (Figure 7). Results from MS however, indicated that β-actin was also present in higher molecular weight regions (Figure 4). However, no β-actin was detected in higher molecular weight areas by this antibody.

**Figure 8:**
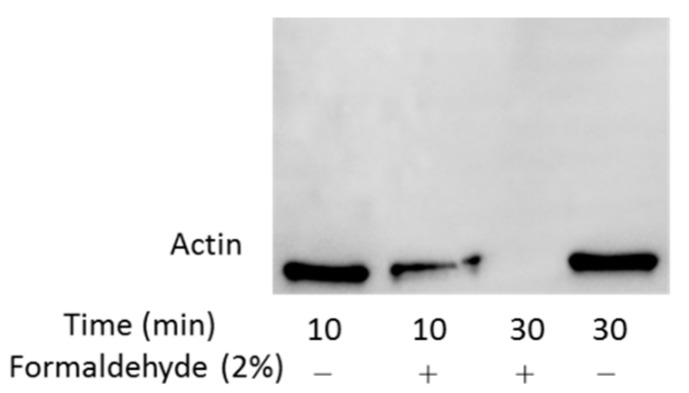
Western blot analysis of β-Actin from the formaldehyde-treated samples. After formaldehyde-induced artificial cross-linking of proteins, actin could not be detected by antibodies any more.

Since two other polyclonal and monoclonal anti-β-actin antibodies were also not able to detect OxPAPC-actin adducts (data not shown), we concluded, that OxPAPC-induced modifications and cross-links of β-actin hampered its recognition by antibodies. Another possible explanation is that the amount of OxPAPC-modified β-actin in high molecular weight regions of gel/blot may be too low for antibody-based detection. To test this possibility, we cross-linked proteins using formaldehyde. Similarly to OxPAPC-treated samples, the antibody could not detect formaldehyde-modified β-actin (Figure 8). After 10 min incubation time the protein amount was decreased, after 30 min the signal was completely lost, but still no β-actin could be detected in higher molecular weight regions (Figure 8). Application of other anti-β-actin antibodies showed the same result (data not shown). We hypothesize that electrophilic molecules either induce irreversible denaturation of protein or modify immunodominant domains of β-actin and, thus, prevent its recognition by antibodies. Thus, although Western blotting cannot detect high molecular weight adducts of actin, the loss of the intact protein upon OxPAPC treatment clearly shows that the protein is modified by OxPAPC thus confirming the findings of MS analysis.

**Figure 9:**
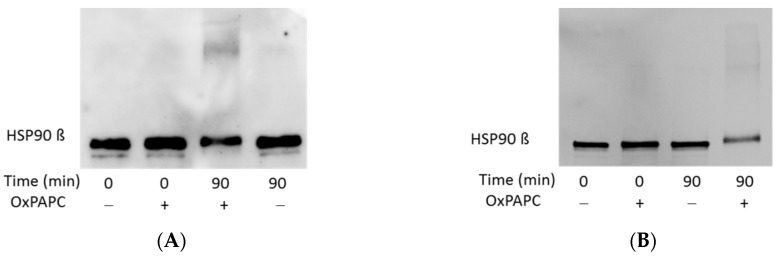
Western blot analysis of HSP90β from the OxPAPC-treated PBS-soluble fractions from two different preparations (**A**,**B**). OxPAPC induces a slight band shift of HSP90β and the generation of high molecular weight protein-OxPAPC complexes. Samples were treated for 0 or 90 min with a final OxPAPC concentration of 0 or 100 µmol/L.

Another protein identified to be modified by OxPAPC using the band shift assay was HSP90β. Using anti-HSP90β antibody we could detect a slight shift of the major band, as well as protein adducts in the higher molecular weight region upon treatment with OxPAPC (Figure 9). These findings are in agreement with the peptide distribution detected by LC-MS/MS.

### 3.4. OxPAPC Modifies Proteins in Live HUVECs

**Figure 10:**
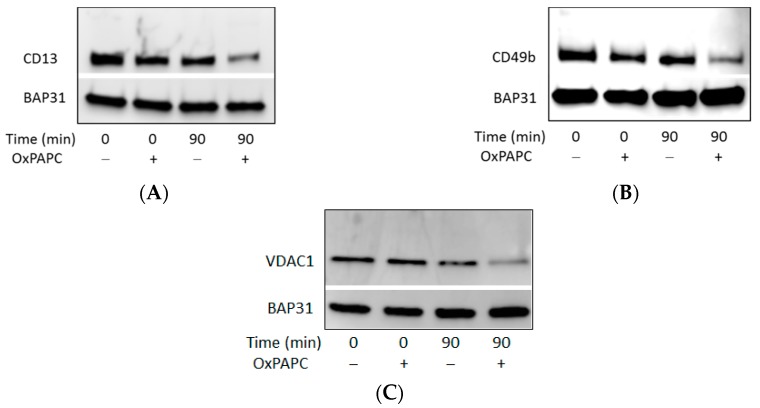
Western blot analysis of (**A**) Aminopeptidase N (CD13), (**B**) Integrin α-2 (CD49b) and (**C**) VDAC-1 after treatment of HUVECs with OxPAPC. Samples were treated for 0 or 90 min with a final OxPAPC concentration of 0 or 80 µmol/L. BAP31: B-cell receptor-associated protein 31 which was identified to not be modified by OxPAPC.

To investigate if identified protein targets of OxPAPC from isolated proteomes are also modified in cell culture, HUVECs were treated with OxPAPC in cell culture medium and subjected to Western blotting analysis. OxPAPC-protein adduct formation was analysed with three different proteins from the RIPA-soluble fraction (Figure 10). Proteins located in the plasma membrane and/or mitochondrial outer membrane, were selected for detection. Similarly to experiments with β-actin, antibodies were not able to detect band shifts in the higher molecular weight region, apparently due to a loss of protein immunoreactivity. However, similarly to β-actin, a decrease of signal intensity was observed for all three proteins after treatment with OxPAPC. These data confirm modification of proteins by OxPAPC and are in agreement with the results of MS analysis.

## 4. Discussion

OxPLs are increasingly recognized as signalling molecules inducing pleiotropic effects in different cell types [6]. Some of the effects (e.g., angiogenesis) depend on electrophilic properties of OxPLs [32] but cannot be explained by simple toxicity of electrophiles. Rather, sub-toxic concentrations of OxPLs seem to regulate specific signal transduction pathways, thus inducing important cellular reactions. OxPL-induced angiogenesis is just one example justifying the need for analysis of proteins that are covalently modified by OxPLs and may be involved in development of (patho-) physiological reactions to these lipids. Such an analysis is not a simple task because (i) oxidation of PUFA-PLs generates a complex mixture of molecular species containing electrophiles with different size, lipophilicity and reactivity; and (ii) cells contain thousands of proteins, some of which may be more prone to oxidation than other. Several methods for identification of OxPL-protein adducts have been published (see Introduction); all of them are based on identification of proteins by mass spectrometry. Although these methods produced important results on cellular targets of electrophilic OxPLs, each procedure has certain limitations. Three of these methods used OxPCs or OxPEs labelled at the head groups with biotin or fluorescent dye [23,24,25,26]. It is apparent that such chemical modification (i) may be more difficult to introduce into other classes of OxPLs and (ii) addition of a bulky group can change the binding characteristics of OxPLs. The method published by Gao et al. [27] is based on chromatographic separation of peptide adducts of OxPLs according to their hydrophobicity before and after cleavage of the fatty acid, which is a highly demanding technique that may be difficult to establish for other types of LOP.

Here we describe a simple method for identification of proteins covalently modified by OxPLs. The method is based on a combination of EMSA with LC-MS/MS (EMSA-MS). The procedure may be characterized as “double-unbiased” because no preliminary information on either oxidized lipids or proteins that are treated with these lipids is needed for the identification of the most reactive protein targets. In other words, this method is independent of the complexity of both the proteome and the LOPs, or from the type of covalent adducts. Furthermore, this approach does not require any kind of chemical tag- or antibody-assisted protein extraction or detection. We demonstrate application of this method for extracts from endothelial cells treated with OxPAPC, but it is to be expected that other types of LOPs and protein mixtures can be analysed using this universal procedure as well. In support of this possibility, we observed band shifts of HUVEC proteins treated with a chemically different LOP, 2-chlorohexadec-15-yn-1-al (Figure S1).

By applying the EMSA-MS to HUVEC lysates, we were able to identify several protein targets of OxPAPC both in soluble and membrane cell fractions (Table 1 and Table 2). It was found that OxPAPC induced not only small shifts in protein band migration, but also the formation of SDS-resistant (supposedly covalent) complexes migrating in high molecular weight regions of the gel (Figure 4). Intermolecular cross-linking is the most probable mechanism generating significantly shifted bands. Since our EMSA analysis used isolated proteomes, any kind of OxPAPC-mediated biological protein modification or changes in protein expression levels as the cause of observed band shifts can be excluded. Comparison of semi-quantitative abundance (emPAI) scores of modified and unmodified proteins showed that the property of proteins to be modified by OxPAPC is not a simple function of their abundance (Figure 5). These results imply that OxPL-protein adducts are not randomly formed and that a certain selectivity of binding is existing. Using Western blot analysis we could prove that selected OxPAPC protein targets identified from isolated proteomes were also modified in live cells treated with OxPAPC (Figure 10).

We found that <5% of detectable proteins from HUVECs demonstrated a band shift after treatment with OxPAPC. In absolute numbers, we found 21 OxPL-modified proteins in the soluble fraction of HUVECs and 37 proteins in RIPA-solubilized membrane fraction. Gugiu et al. have analysed binding of OxPLs to proteins in human aortic endothelial cells using a different detection principle, namely biotin-labelled OxPAPE [23]. Twenty-eight modified proteins were identified, which is close to the number found in our work. Interestingly, nine proteins were also found in our samples (Table 1 and Table 2). Stemmer et al. used fluorescently labelled OxPAPC components POVPC and PGPG and found >50 modified proteins in mouse macrophage cell line [26]. From these, nine proteins were also identified in our study (Table 1 and Table 2). In summary, comparison of our results with two published studies on cells treated with OxPLs points to a similar sensitivity of the methods (dozens of modified proteins were identified in each cell type). Partial, but significant, overlap of our hits with those obtained using independent methods supports the reliability of results generated by EMSA-MS. Furthermore, using STRING analysis, we observed enrichment of proteins related to (i) cytoskeleton; (ii) protein folding; and (iii) mitochondria (Figure 6). All these are known as common themes in electrophile-modified proteome, thus further supporting the validity of the EMSA-MS method [11,15,20,33,34,35,36,37,38,39,40].

Similarly to other methods described above, our technique has limitations. Since the method does not identify exact structures of lipids modifying amino acids, false positive hits (i.e., proteins modified by chemically different LOPs) may arise due to transformation of added LOPs to their reactive derivatives, or due to generation of reactive oxygen species in the course of incubation. However, because the ‘target’ LOP is present in large molar excess as compared to its secondary products generated in the course of incubation, the secondary products can only have a limited impact on the whole electrophile-modified proteome and false positive results are unlikely to significantly bias the results. On the other hand, false negative results are also possible. Certain modifications by LOPs can have no effect on protein electrophoretic mobility and, therefore, some modified proteins can be falsely characterized as non-modified. Further studies are needed in order to understand how lipid adduction changes protein mobility. It seems likely that the method predominantly identifies multiply modified protein. This has certain advantages, because such proteins may be especially sensitive to LOPs and, as a consequence, play a mechanistic role in oxidative stress-induced damage. In other words, although the lipid specificity of EMSA-MS is not investigated, the method may be especially helpful for identification of proteins that are most sensitive to LOPs. Thus, the major application of the EMSA-MS technique is a search for protein targets of LOPs with the following mechanistic analysis of their role in oxidation-induced pathology.

An unexpected finding done in this work is that modification with OxPLs strongly inhibited recognition of proteins by standard commercial antibodies. OxPAPC-induced cross-linked high molecular weight complexes detected by MS could only be immunostained in case of HSP90β (Figure 9). Slight band shifts were observed on Western blots of modified β-actin and HSP90β (Figure 7 and Figure 9). For all other analysed proteins, only a distinct decrease of signal intensity was observed by Western blotting after treatment with OxPAPC (Figure 10). Since artificial cross-linking using formaldehyde did not improve detection of high molecular weight adducts of actin, insufficient actin amount in shifted bands as the cause of the lack of signal in high molecular weight areas of gel can be excluded. We hypothesize that electrophilic molecules may induce irreversible denaturation of proteins that prevents partial refolding of SDS-treated proteins on blotting membranes and, thus, prevent binding of antibodies that recognize 3-D epitopes. Alternatively, for antibodies recognizing linear epitopes, immunodominant domains of proteins may be covalently modified by LOPs thus preventing their recognition by antibodies. Independently of the mechanism(s) of immunoreactivity loss, our data suggest that (i) Western blotting cannot be recommended for detection of band shifts induced by lipid adduction to proteins and (ii) β-actin should be used with caution as a housekeeping gene in experiments where the cells are exposed to oxidative stress.

In summary, here we describe a novel combination of electrophoretic mobility shift assay with MS-based protein sequencing, which allows identification of proteins modified by LOPs without an a priori knowledge of reactive lipid species or target proteins. We applied this method to endothelial cells and identified several known, but also many new, OxPAPC-binding proteins. We expect that this technically relatively simple method can be widely applied for label-free analysis of lipid-protein interaction in variable protein samples treated with different LOPs.

## Figures and Tables

**Table 1:** Potential OxPAPC target proteins identified from the PBS-soluble fraction.

Protein	Accession	Reported OxPL Target *
14-3-3 protein theta	1433T_HUMAN	
40S ribosomal protein SA	RSSA_HUMAN	
60 kDa heat shock protein, mitochondrial	CH60_HUMAN	
78 kDa glucose-regulated protein	GRP78_HUMAN	[23,26]
Actin, cytoplasmic 1	ACTB_HUMAN	[23]
Chloride intracellular channel protein 1	CLIC1_HUMAN	
Elongation factor 1-gamma	EF1G_HUMAN	
Elongation factor 2	EF2_HUMAN	
Endoplasmin	ENPL_HUMAN	[26]
Glyceraldehyde-3-phosphate dehydrogenase	G3P_HUMAN	
Heat shock protein HSP 90-beta	HS90B_HUMAN	
L-lactate dehydrogenase A chain	LDHA_HUMAN	
L-lactate dehydrogenase B chain	LDHB_HUMAN	
Myosin-9	MYH9_HUMAN	[23]
Protein-glutamine gamma-glutamyltransferase 2	TGM2_HUMAN	
Puromycin-sensitive aminopeptidase	PSA_HUMAN	
Pyruvate kinase PKM	KPYM_HUMAN	[26]
Spectrin alpha chain, non-erythrocytic 1	SPTN1_HUMAN	
Tubulin alpha-1A chain	TBA1A_HUMAN	
Tubulin beta chain	TBB5_HUMAN	[23]
Vimentin	VIME_HUMAN	[23,26]

* These proteins were identified to by targets of oxidized phospholipids (OxPLs) by proteomic based studies using comparable OxPLs and endothelial cells [23] or macrophages [26]. OxPAPC, oxidized palmitoyl-arachidonoyl-phosphatidylcholine; PBS, phosphate buffered saline.

**Table 2:** Potential OxPAPC target proteins identified from the PBS-insoluble/RIPA-soluble fraction.

Protein	Accession	Reported OxPL Target *
ADP/ATP Translocase 2	ADT2_HUMAN	
Aminopeptidase N	AMPN_HUMAN	
ATP synthase F(0) complex subunit B1	AT5F1_HUMAN	
ATP synthase subunit alpha, mitochondrial	ATPA_HUMAN	[23]
Brain acid soluble protein 1	BASP1_HUMAN	
Coiled-coil domain-containing protein 47	CCD47_HUMAN	
Cytoskeleton-associated protein 4	CKAP4_HUMAN	[23]
Endoplasmin	ENPL_HUMAN	[26]
Erlin 1	ERLN1_HUMAN	
Fatty aldehyde dehydrogenase	AL3A2_HUMAN	
Flotillin 1	FLOT1_HUMAN	
GTPase IMAP family member 1	GIMA1_HUMAN	
Guanine nucleotide-binding protein G (i) subunit alpha-2	GNAI2_HUMAN	[23]
Guanine nucleotide-binding protein G(I)/G(S)/G(T) subunit beta-1	GBB1_HUMAN	[26]
Guanine nucleotide-binding protein G(I)/G(S)/G(T) subunit beta-2	GBB2_HUMAN	
Inhibitor of nuclear factor kappa-B kinase-interacting protein	IKIP_HUMAN	
Integrin alpha-2	ITA2_HUMAN	
Leucine-rich repeat-containing protein 59	LRC59_HUMAN	
Myoferlin	MYOF_HUMAN	
NADH-cytochrome b5 reductase 3	NB5R3_HUMAN	
NADH dehydrogenase [ubiquinone] 1 alpha subcomplex subunit 9	NDUA9_HUMAN	
Perilipin 3	PLIN3_HUMAN	
Polypeptide N-acetylgalactosaminyltransferase 1	GALT1_HUMAN	
Polypyrimidine tract-binding protein 1	PTBP1_HUMAN	
Prohibitin	PHB_HUMAN	
Prohibitin 2	PHB2_HUMAN	[26]
RNA-binding protein Raly	RALY_HUMAN	
Serine/arginine-rich splicing factor 1	SRSF1_HUMAN	
Serine/arginine-rich splicing factor 9	SRSF9_HUMAN	
Sideroflexin-3	SFXN3_HUMAN	
Trifunctional enzyme subunit alpha, mitochondrial	ECHA_HUMAN	
Trifunctional enzyme subunit beta, mitochondria	ECHB_HUMAN	[23]
Vesicle-trafficking protein SEC22b	SC22B_HUMAN	
Voltage-dependent anion-selective channel protein 1	VDAC1_HUMAN	[26]
Voltage-dependent anion-selective channel protein 2	VDAC2_HUMAN	[26]
X-ray repair cross-complementing protein 6	XRCC6_HUMAN	
60S ribosomal protein L19	RL19_HUMAN	

* These proteins were identified to by targets of OxPLs by proteomic based studies using comparable OxPLs and endothelial cells [23] or macrophages [26].

## Supplementary Materials

The following are available online at http://www.mdpi.com/2076-3921/7/9/116/s1, Figure S1 Gel band shift analysis of PBS-soluble HUVEC proteins. Figure S2 Selected MS/MS spectra of VDAC-1. Figure S3 Selected MS/MS spectra of Aminopeptidase N. Figure S4 Selected MS/MS spectra of HSP90β. Figure S5 Selected MS/MS spectra of Integrin alpha-2. Figure S6 Selected MS/MS spectra of ADP/ATP translocase 2.

## Abbreviations

BAP31	B-cell receptor-associated protein 31
emPAI	Exponentially Modified Protein Abundance Index
EMSA	Electrophoretic mobility band shift analysis
HNE	4-hydroxynonenal
HRP	horseradish peroxidase
HSP90β	heat shock protein HSP 90-beta
HUVEC	human umbilical vein endothelial cell
KODA-PC	9-keto-12-oxo-10-dodecenoate-phosphatidylcholine
LOP	lipid oxidation product
MS	mass spectrometry
OxPAPC	oxidized palmitoyl-arachidonoyl-phosphatidylcholine
oxPL	oxidized phospholipid
PAPC	palmitoyl-arachidonoyl-phosphatidylcholine
PAPE	1-palmitoyl-2-arachidonoyl-*sn*-glycero-3-phosphoethanolamine
PAPS	1-palmitoyl-2-arachidonoyl-*sn*-glycero-3-phospho-l-serine
PBS	Phosphate-buffered saline
PC	phosphatidylcholine
PE	phosphoethanolamine
PBS-PI	phosphate buffered containing protease inhibitor cocktail
RIPA buffer,	Radioimmunoprecipitation assay buffer
PL	phospholipid
PLPC	1-palmitoyl-2-linoleoyl-*sn*-glycero-3-phosphatidylcholine
PUFA	polyunsaturated fatty acid
SDS	sodium dodecyl sulfate
VDAC-1	voltage-dependent anion-selective channel protein 1
