# Supplementary material for: Unbiased Identification of Proteins Covalently Modified by Complex Mixtures of Peroxidized Lipids Using a Combination of Electrophoretic Mobility Band Shift with Mass Spectrometry
Source: Antioxidants (Basel). 2018 Aug 30;7(9):116. doi: 10.3390/antiox7090116 (PMC6162613; doi:10.3390/antiox7090116)
Supplement: Supplementary file 1 [file antioxidants-07-00116-s001.docx]

**Supplementary Material**

**1. Reagents**

Unless otherwise specified, all reagents were from Sigma-Aldrich (St. Louis, MO, USA).

*1.1. Reagents for the Isolation of HUVEC Proteomes*

Phosphate-buffered saline (137 mmol/L NaCl, 2.7 mmol/L KCl, 10 mmol/L Na_2_HPO_4_, 1.8 mmol/L KH_2_PO_4_) was prepared in-house and supplemented with cOmplete™ Protease Inhibitor Cocktail (PBS-PI).

RIPA buffer was composed of 150 mmol/L NaCl, 1.0% (*v*/*v*) IGEPAL® CA-630, 0.5% (w/v) sodium deoxycholate, 0.1% (*w*/*v*) SDS, 50 mmol/L Tris, pH 8.0, and was supplemented with cOmplete™ Protease Inhibitor Cocktail according to the manufacturer's instructions (RIPA-PI).

*1.2. Reagents for EMSA*

Laemmli buffer (4×, 250 mmol/L Tris-HCl, pH 6.8, 40% (*v*/*v*) glycerol, 8% (*w*/*v*) SDS, 0.02% (*w*/*v*) bromophenol blue, and 10% (*v*/*v*) 2-mercaptoethanol) was obtained from Bio-Rad Laboratories (Hercules, CA, USA).

Tris/glycine/SDS running buffer (25 mmol/L Tris, 192 mmol/L glycine, and 0.1% (*w*/*v*) SDS, pH 8.3) was obtained from Bio-Rad Laboratories (Hercules, CA, USA).

Homemade 10% acrylamide gels were cast as described previously [1]. The separating gel was composed of 10% (*v*/*v*) acrylamide/bis-acrylamide (37.5:1), 375 mmol/L Tris-HCl, pH 8.8, 0.1% (*w*/*v*) SDS, 0.033% (*w*/*v*) ammonium persulfate, 0.066% (*v*/*v*) tetramethylethylenediamine.The stacking gel was composed of 3.9% (*v*/*v*) acrylamide/bis-acrylamide (37.5:1), 125 mmol/L Tris-HCl, pH 6.8, 0.1% (*w*/*v*) SDS, 0.05% (*w*/*v*) ammonium persulfate, and 0.1% (*v*/*v*) tetramethylethylenediamine.

*1.3. Reagents for In-Gel Protein Digestion and Nano-LC-MS/MS Analysis*

Water (HPLC grade) and acetonitril (HPLC grade) were obtained from VWR (Radnor, USA). Dithiothreiotol and iodoacetamide were dissolved in 50 mmol/L ammonium hydrogen carbonate (LC-MS grade) to a final concentration of 10 mmol/L and 50 mmol/L, respectively. Trypsin (sequencing grade, Roche, Basel, Switzerland) was dissolved in 50 mmol/L ammonium hydrogen carbonate to a final concentration of 5 ng/L.

**
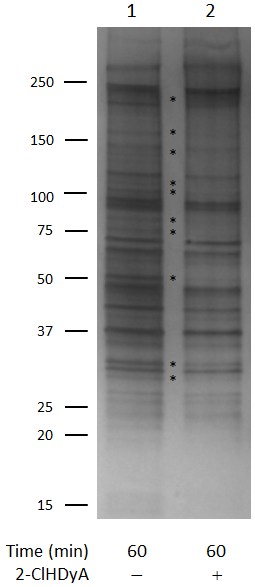
**

**Figure S1.** Gel band shift analysis of PBS-soluble HUVEC proteins. 2-chlorohexadec-15-yn-1-al (2-ClHDyA) influences the electrophoretic mobility of proteins (lane 2) compared to control samples (lane 1). The final 2-ClHDyA concentration in treated samples was 100 µmol/L. Visible changes are marked with asterisks.


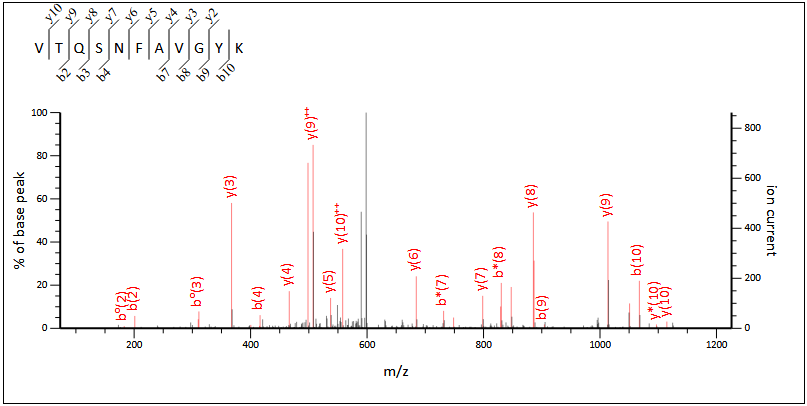


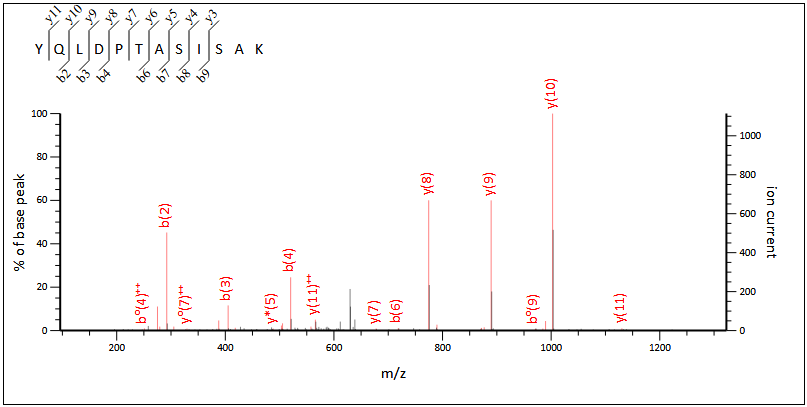


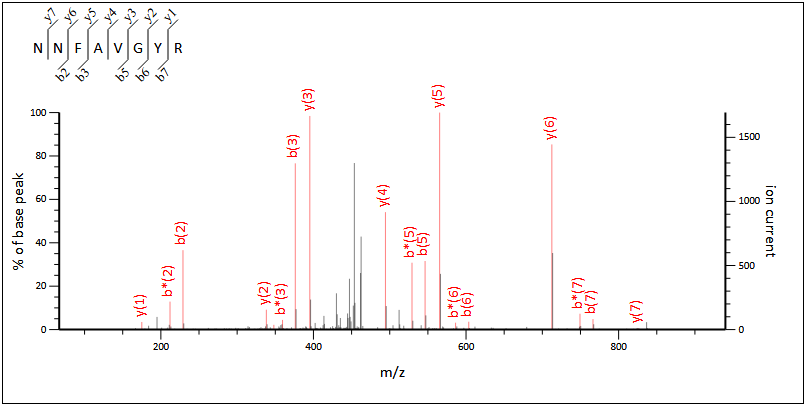


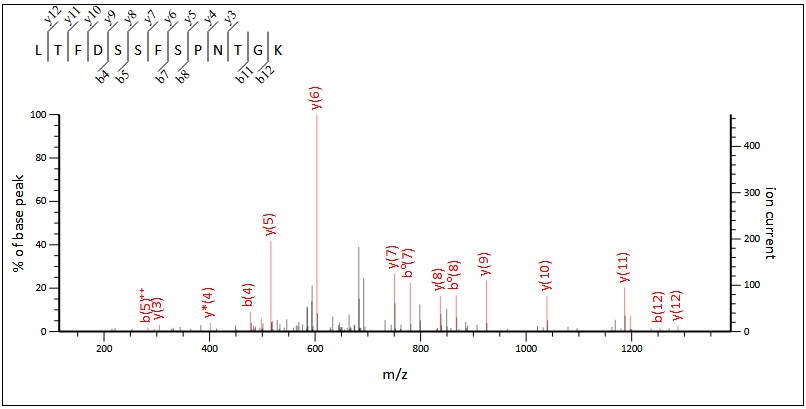


**Figure S2.** Selected MS/MS spectra of VDAC-1.

**
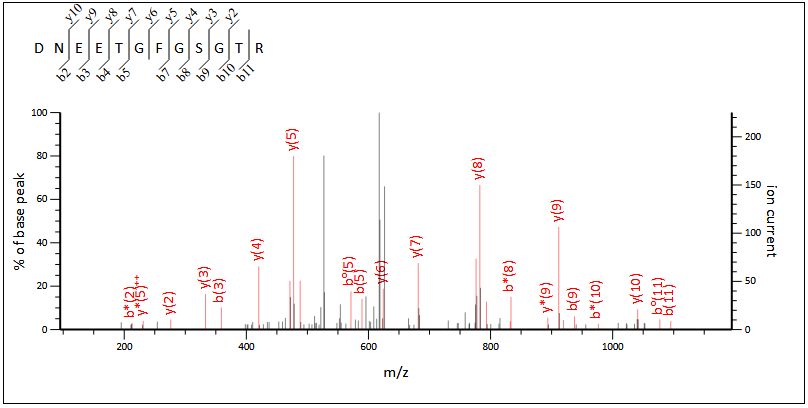
**

**
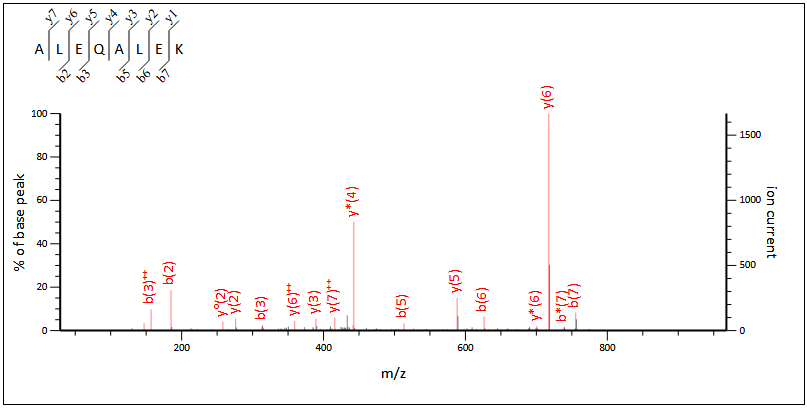
**

**
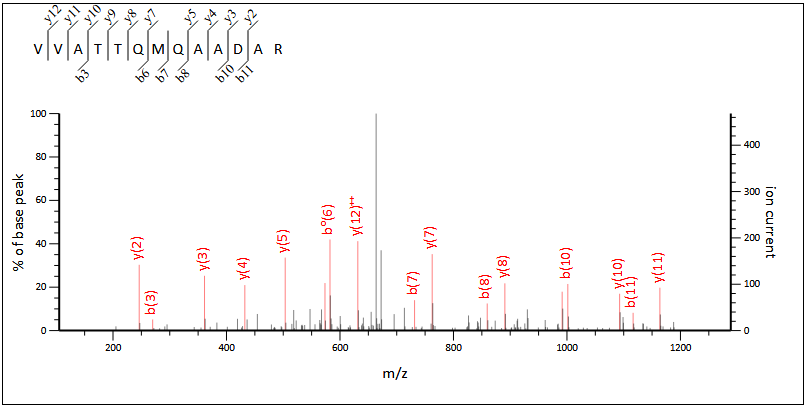
**

**
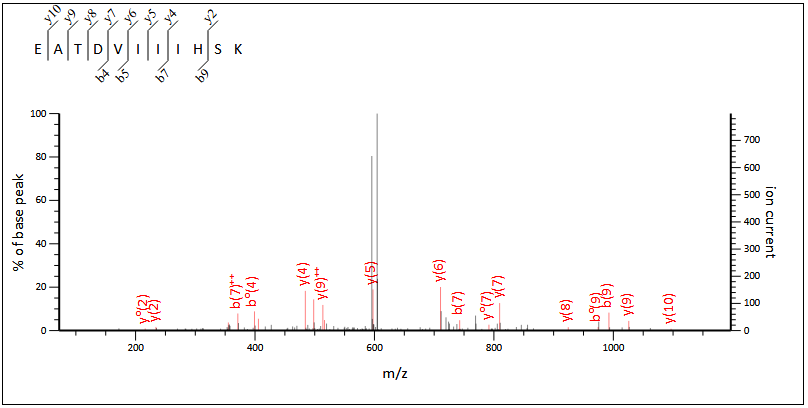
**

**Figure S3.** Selected MS/MS spectra of Aminopeptidase N.

**
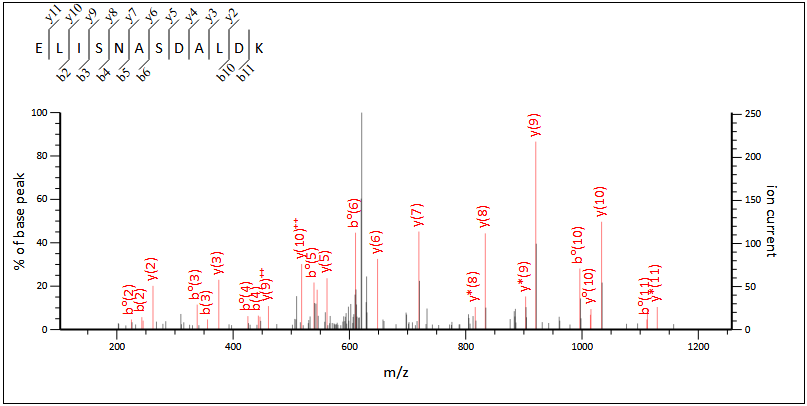
**

**
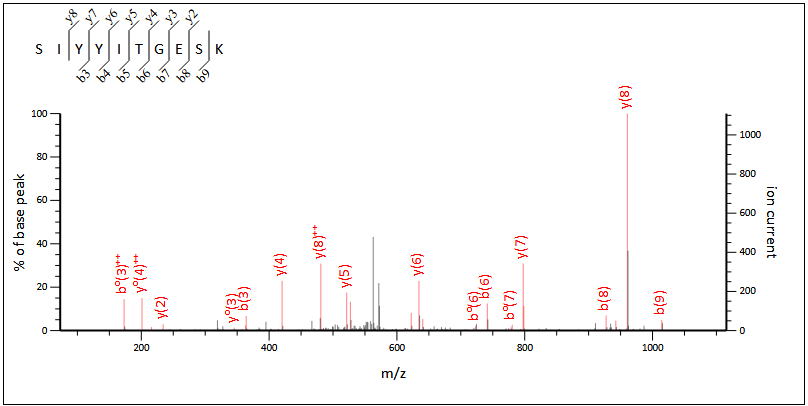
**

**
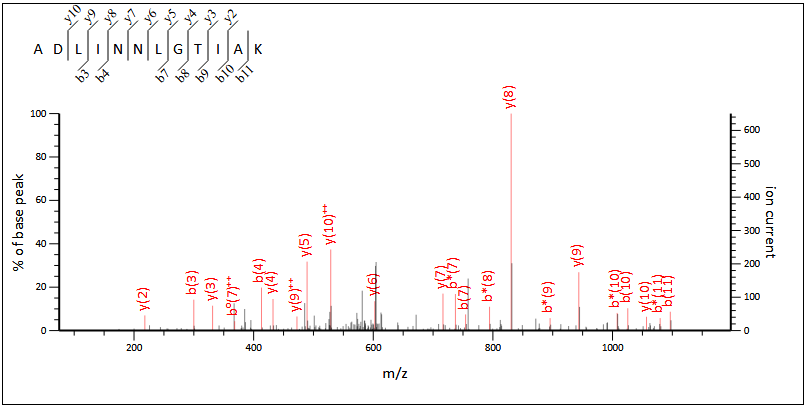
**

**
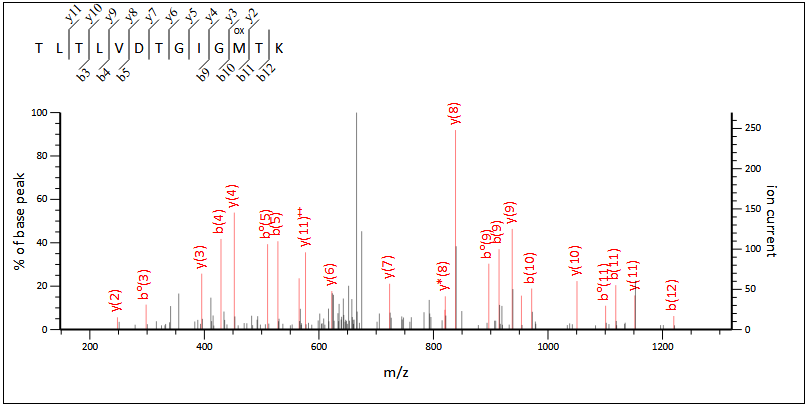
**

**Figure S4.** Selected MS/MS spectra of HSP90β.


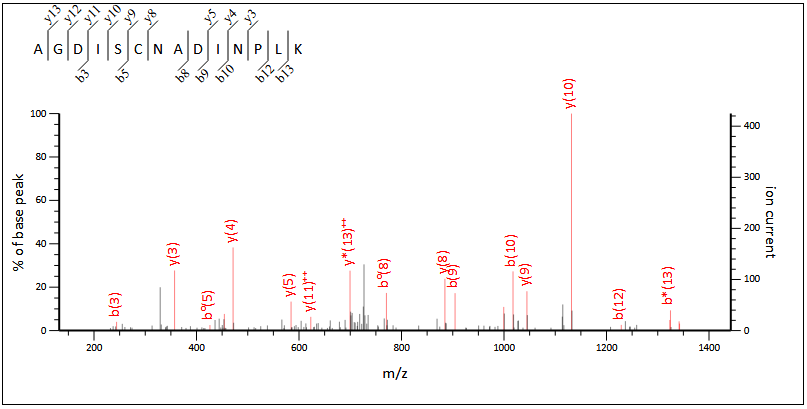


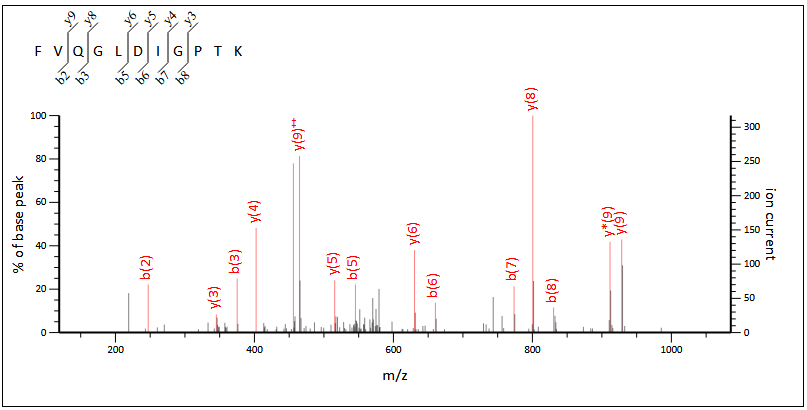


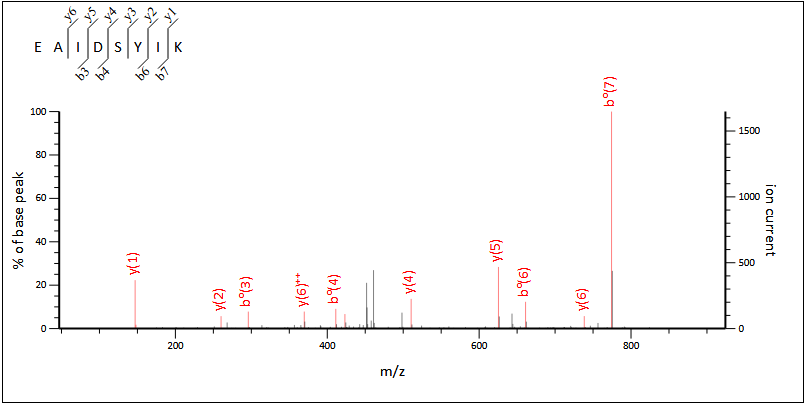


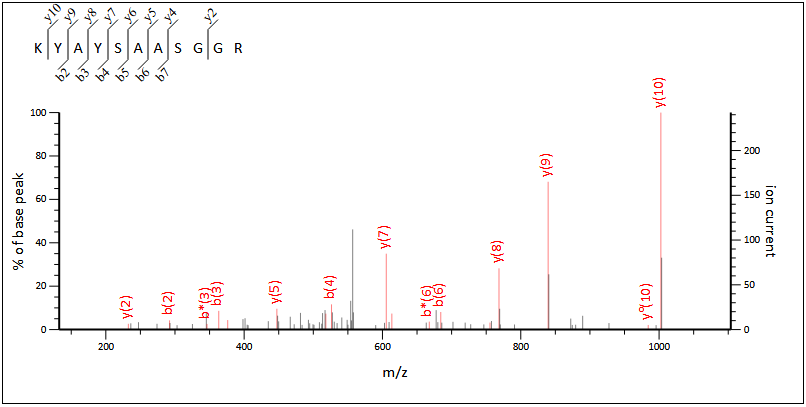


**Figure S5.** Selected MS/MS spectra of Integrin alpha-2.

**
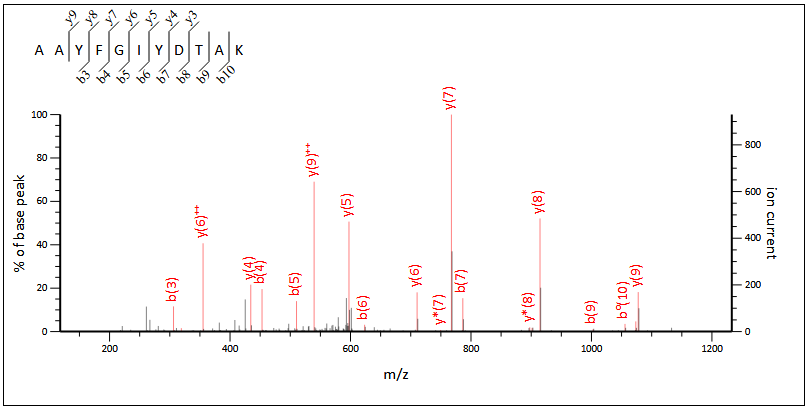
**

**
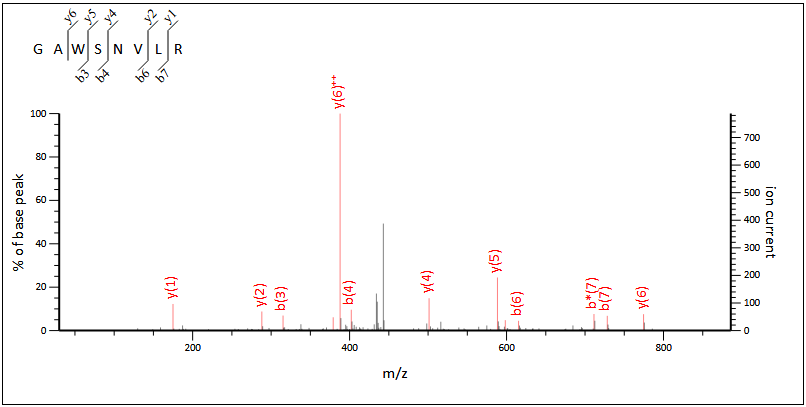
**

**
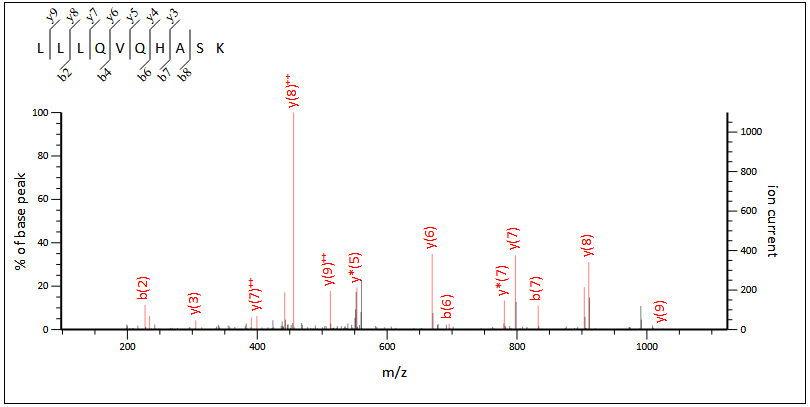
**

**
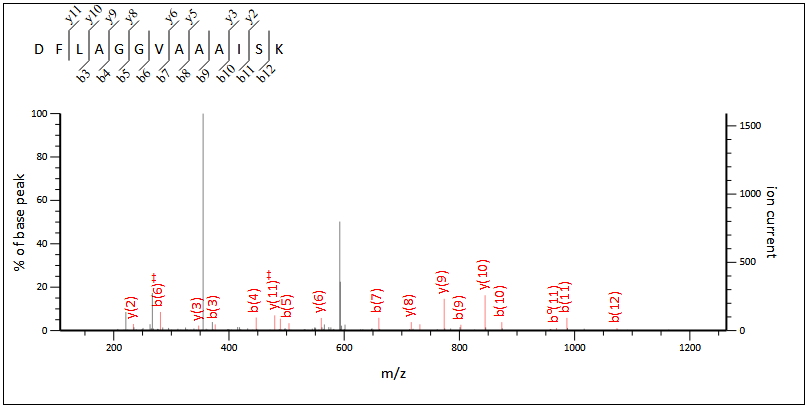
**

**Figure S6.** Selected MS/MS spectra of ADP/ATP translocase 2.

**References**

1 Gallagher SR. One-dimensional SDS gel electrophoresis of proteins. Curr Protoc Protein Sci. 2012 Apr;Chapter 10:Unit 10.1.1-44.
